# Proteome Analysis of Rice (*Oryza sativa* L.) Mutants Reveals Differentially Induced Proteins during Brown Planthopper (*Nilaparvata lugens*) Infestation

**DOI:** 10.3390/ijms14023921

**Published:** 2013-02-15

**Authors:** Jatinder Singh Sangha, H. Chen Yolanda, Jatinder Kaur, Wajahatullah Khan, Zainularifeen Abduljaleel, Mohammed S. Alanazi, Aaron Mills, Candida B. Adalla, John Bennett, Balakrishnan Prithiviraj, Gary C. Jahn, Hei Leung

**Affiliations:** 1 Plant Breeding, Genetics and Biochemistry Division, International Rice Research Institute, DAPO Box 7777, Metro Manila, Philippines; E-Mails: jatinder.sangha@dal.ca (J.S.S.); yolanda.chen@uvm.edu (Y.H.C.); Johnpiabennett@yahoo.com (J.B.); gjahnster@gmail.com (G.C.J.); 2 Department of Environmental Sciences, Faculty of Agriculture, Dalhousie University, Truro, Nova Scotia B2N 5E3, Canada; E-Mails: jkaur@nsac.ca (J.K.); bprithiviraj@nsac.ca (B.P.); 3 Department of Plant and Soil Sciences, University of Vermont, 63 Carrigan Drive, Burlington, VT 05405, USA; 4 Genome Research Chair Unit, Biochemistry Department, College of Science, King Saud University, PO Box 2455, Riyadh 11451, Saudi Arabia; E-Mails: wkhan@ksu.edu.sa (W.K.); zarifeen@ksu.edu.sa (Z.A.); msanazi@ksu.edu.sa (M.S.A.); 5 Crops and Livestock Research Center, Agriculture and Agri-Food Canada, 440 University Ave., Charlottetown, Prince Edward Island C1A4N6, Canada; E-Mail: millsaaron@gmail.com; 6 Department of Entomology, College of Agriculture, University of the Philippines, Los Banos, Laguna 4031, Philippines; E-Mail: aydsadalla@yahoo.com; 7 Georgetown University Medical Center, Department of Microbiology and Immunology, Washington, DC 20057, USA

**Keywords:** rice resistance, brown planthopper, proteomics, S-like RNase, molecular docking

## Abstract

Although rice resistance plays an important role in controlling the brown planthopper (BPH), *Nilaparvata lugens*, not all varieties have the same level of protection against BPH infestation. Understanding the molecular interactions in rice defense response is an important tool to help to reveal unexplained processes that underlie rice resistance to BPH. A proteomics approach was used to explore how wild type IR64 and near-isogenic rice mutants with gain and loss of resistance to BPH respond during infestation. A total of 65 proteins were found markedly altered in wild type IR64 during BPH infestation. Fifty-two proteins associated with 11 functional categories were identified using mass spectrometry. Protein abundance was less altered at 2 and 14 days after infestation (DAI) (T1, T2, respectively), whereas higher protein levels were observed at 28 DAI (T3). This trend diminished at 34 DAI (T4). Comparative analysis of IR64 with mutants showed 22 proteins that may be potentially associated with rice resistance to the brown planthopper (BPH). Ten proteins were altered in susceptible mutant (D1131) whereas abundance of 12 proteins including S-like RNase, Glyoxalase I, EFTu1 and Salt stress root protein “RS1” was differentially changed in resistant mutant (D518). S-like RNase was found in greater quantities in D518 after BPH infestation but remained unchanged in IR64 and decreased in D1131. Taken together, this study shows a noticeable level of protein abundance in the resistant mutant D518 compared to the susceptible mutant D1131 that may be involved in rendering enhanced level of resistance against BPH.

## 1. Introduction

Plants resist herbivorous insects through a combination of constitutive or induced defenses that are generally manifested through poor feeding, abnormal development, low fecundity or even mortality. Various molecular and biochemical approaches can be used to determine the role of constitutive or induced plant defense responses against herbivory [1–3]. These approaches are equally useful to reveal complex plant-insect interactions that may assist in identification of candidate genes involved in plant defense response [4,5].

Rice is susceptible to a number of insect pests that affect its yield and quality; consequently, several modern rice varieties have so far selectively been developed with resistance to insect pests [6]. Resistant varieties differ considerably in their responses to guard against pests particularly due to the presence of resistant (R) genes. For instance, rice varieties may be bred with R genes for resistance to stem borers, planthoppers or a combination of genes for resistance against multiple pests. Nevertheless, the induction of plant defense mechanisms that includes the production of nutritional and defensive proteins, phenolic compounds or protease-inhibitors and so will strongly contribute towards protecting the plants against insect damage [4,7,8]. Although the presence of R genes potentiates rice defense mechanisms against herbivores, the role of other non-R gene like mechanisms and their mutual interaction with R genes during herbivory cannot be excluded [6–9]. Broadly speaking, the overall resistance to insect infestation will be a cumulative response of different cellular processes in the plant, including input of R and non-R genes that may be interacting particularly during stress to help the plant express their defense response. Elucidating the complex phenomena of rice defense is will be important to plan rice resistance strategies for existing and emerging pests.

The brown planthopper (BPH), *Nilaparvata lugens* Stål (Hemiptera: Delphacidae), is a secondary pest of rice and causes significant economic loss to susceptible rice cultivars [10,11]. Continuous feeding by BPH populations for several days on rice in the field may lead to hopperburn, a condition resulting from wilting of tillers [9]. Growing resistant varieties of rice is considered the most effective and environment friendly way to control the BPH. So far, more than 20 rice genes and quantitative trait loci (QTLs) have been identified and introduced to various cultivars through breeding in order to confer BPH resistance [11,12]. Rice resistance through the introduction of QTLs has been shown to be effective against BPH [13]. However, due to the genetic complexity between resistant rice cultivars, it has been difficult to explain the function QTLs play in the resistance mechanisms against BPH that further hinders the performance of resistance cultivars in different environments. Expression analysis of global genes and proteins is one strategy to understand molecular responses of rice plants during BPH stress to elucidate how different genes and proteins involve and interact during defense activities and help their selection for use in breeding rice resistance against BPH.

Rice defense against BPH has been well documented and the factors involved in rice resistance against BPH are usually associated with the differential regulation of genes and proteins during infestation [7,10,11,14,15]. Many studies revealed physiological and metabolic changes in rice plants during BPH feeding [4,7–11]. Such alterations in rice plant with BPH infestation also accompany transcriptional activation or repression of plant genes and reorganization of the gene expression profile during stress [7,8,14]. It seems that not only the genes associated with cell defense are induced by BPH, genes that are involved in plant metabolism are also altered possibly through reallocation of necessary metabolites required for growth, reproduction, and storage towards defense activities instead [11]. In this process, the genes associated with abiotic stress, pathogen stress and signaling pathways are reduced, whereas photosynthesis and defense related genes are increased [7,8,14]. Extensive expression analysis of genes and proteins has facilitated the identification of several distinct genes affected by BPH feeding in rice that helped to differentiate susceptible *vs.* resistant rice cultivars [9,11,15–17]. For example, 160 unique genes were identified that responded to BPH infestation [15]. Similarly, proteomics approach differentiated a susceptible line from a resistant line carrying a resistance gene BPH15 and identified additional eight genes differentially expressed in rice with BPH infestation [9]. Advances in these tools and the ability to differentiate plant reaction to BPH stress suggests for a significant role expression analysis can play in developing rice resistance to BPH.

Mutational approach can play significant role in identifying proteins involved in rice response under specific physiological conditions such as abiotic and biotic stress [18]. A comparative proteome analysis involving wild type rice and the mutants revealed contrasting differences in proteins induced in contrasting genotypes [19,20]. Rice blast lesion mimic mutant (*blm*) was differentiated from wild type plants based on pathogenesis-related class 5 and 10 proteins including a novel OsPR10d protein specific to the mutants’ response. This study also reported increase in phytoalexins and oxidative stress related marker proteins in *blm* mutant [20]. In another study, more than 150 protein spots were identified as differentially regulated between normal leaves of wild type and spotted leaves of the spl6 rice mutant, indicating the potential of proteomics to elucidate molecular response of rice [21]. Proteomics of rice mutants, will certainly help to elucidate different proteins potentially involved in rice interaction with BPH and explain rice defense strategies against biotic stress [22] This approach could be useful to explore QTL dependent resistance in rice cultivars such as IR64 and its mutants. IR64 is a modern rice variety developed at International Rice Research Institute (IRRI) that carries the major gene Bph1 and other minor genes located in a QTL responsible for resistance to BPH. The durable nature of BPH resistance in IR64 is thought to be due to synergy with minor genes, which contribute to a combined resistance through the mechanisms of antixenosis, antibiosis and tolerance [13]. The mutants of this cultivar have been developed at IRRI [23] and used for elucidating various physiological responses of rice.

The objective of the present study is to describe the proteomic responses of indica rice IR64 and two of its chemically generated mutants, one resistant and one susceptible to BPH infestation. Previous study with these IR64 mutants found no growth or yield penalty under normal field conditions [23]. The contrasting phenotypes expressed by mutants that are essentially near-isogenic offer an opportunity to perform genetic analysis in response to BPH infestation and identify specific genes or proteins related to rice resistance. We performed a time-series analysis of gradual BPH stress on IR64 to identify BPH induced proteins. These proteins were further compared between wild type IR64 and the mutants to explain potential role of differentially altered proteins with BPH infestation.

## 2. Results

### 2.1. Rice Phenotype during BPH Stress

**Figure 1:**
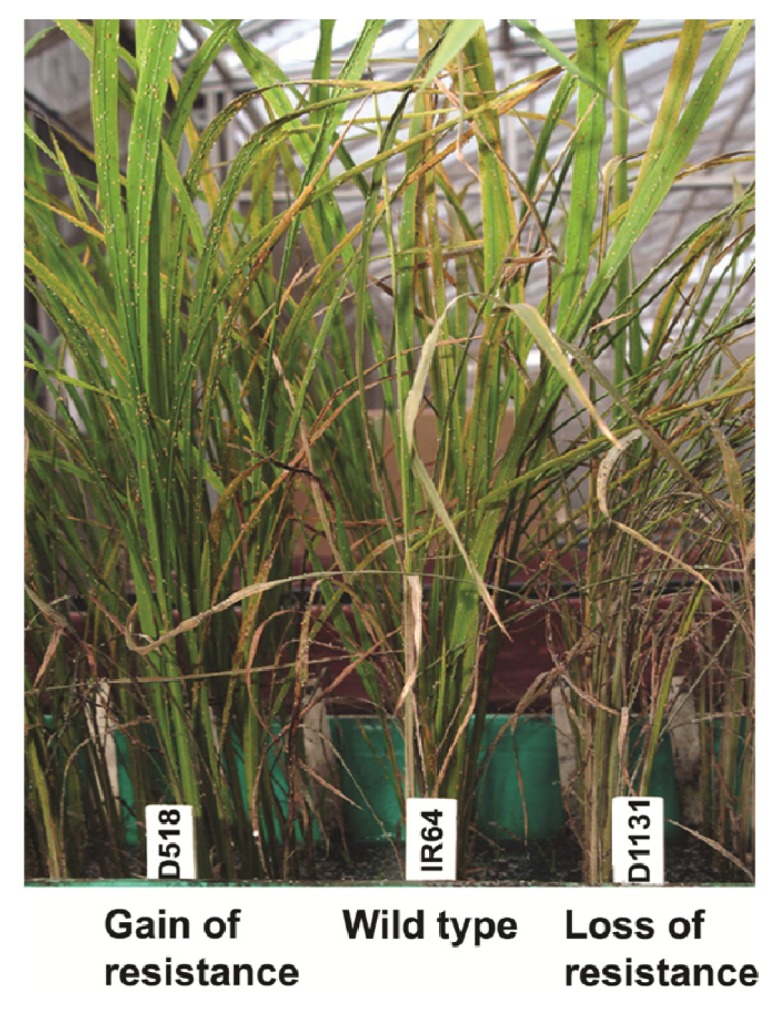
Phenotype of wild type IR64 and mutant plants exposed to brown planthopper (*N. lugens)* infestation under greenhouse conditions during seedbox screening (free choice). Pre-germinated seeds were sown in the heat sterilized soil in seed boxes a density of 15 seedlings per row. Hopperburn symptoms appeared first on D1131, followed by IR64 and lastly on D518. The experiment was repeated 3 times.

Using a modified seedbox screening technique [13] ten-day-old seedlings were uniformly infested with 3–4 second-instar BPH nymphs with free choice to settle on their preferred host. Hopperburn symptoms were observed at different intervals (Table 1). Following infestation, continuous feeding by growing second generation BPH nymphs caused wilting of the seedlings, leading to hopperburn (browning of stem and leaves) symptoms first on D1131, followed by IR64 and finally on D518 (Figure 1). Early on infestation (T1 and T2), damage symptoms were not detected on infested plants. This is likely due to a low number of nymphs that were initially released on plants, which did not cause enough damage and plants were able to overcome low level of insect stress. The difference in phenotype among the mutants and IR64 was more obvious at T3 and T4 (28 DAI and 34 DAI, respectively). The average leaf damage rate was recorded on a modified 1–9 scale (1 = resistant, 9 = highly susceptible) [23]. Leaf damage at T3 was lowest for D518 (3.5), intermediate for IR64 (5.2), and highest for D1131 (6.8).

### 2.2. Proteome Analysis of BPH Induced Proteins in IR64

**Figure 2:**
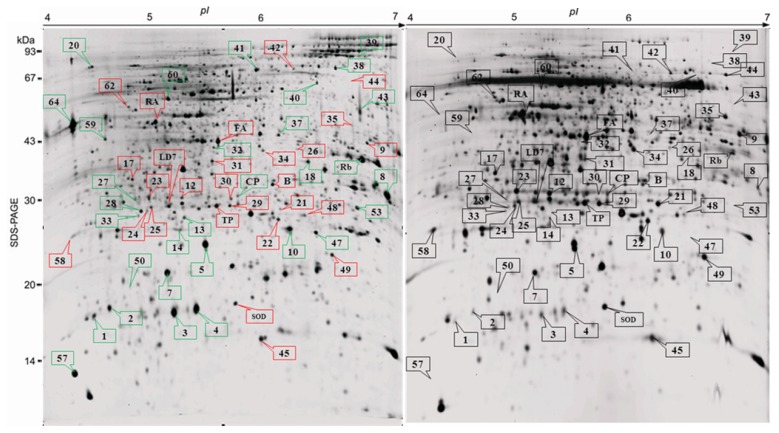
2-D gel electrophoresis of IR64 leaf sheath proteins following brown planthopper (*N. lugens*) infestation (left panel) and control (right panel) condition. Total plant proteins extracted using TCA-Acetone method were separated on 15% SDS PAGE using non linear (NL) 18-cm IPG strips. The gels were stained with silver nitrate for protein detection. The red boxes represent down regulated proteins whereas green boxes represent up regulated proteins after BPH infestation.

**Figure 3:**
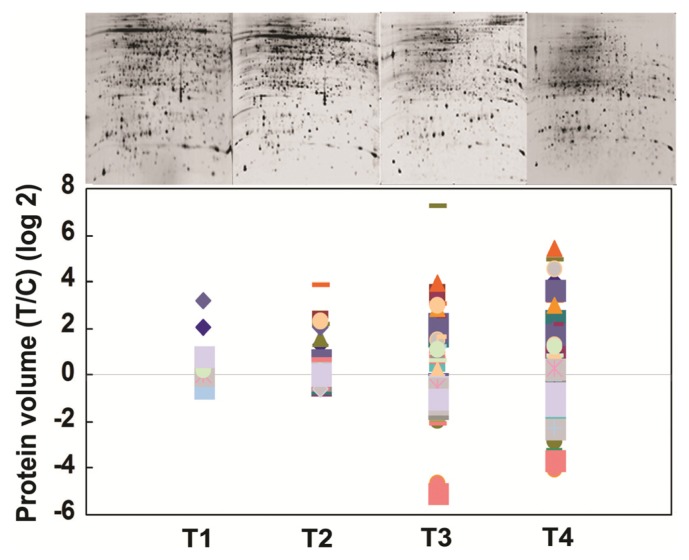

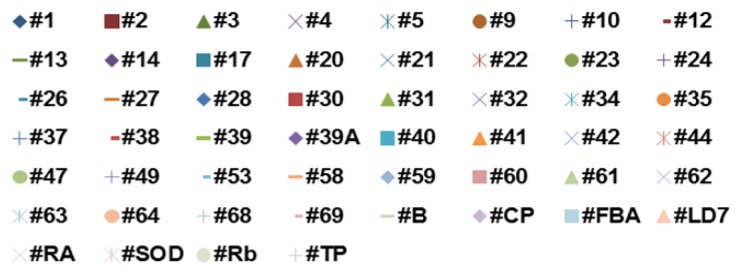
Abundance of brown planthopper (*N. lugens*) responsive proteins in IR64 at different days after BPH infestation (DAI) (T1 = 2 DAI; T2 = 13 DAI; T3 = 28 DAI; T4 = 34 DAI). The figure shows log_2_ values of proteins [BPH infested (T)/control (C)] at different time points. (*n* = 3; *p* < 0.05). The protein legends in the figure represent induction response of IR64 proteins (log 2 value) after BPH infestation

The proteome response of wild type IR64 during BPH infestation over 5-week period after infestation was first studied. This is a condition that simulates natural infestation on rice under field conditions. Among 1500 protein spots visualized on silver stained 2-D polyacrylamide gel (3–10 pH), 65 protein spots were found altered (*p* < 0.001) with BPH infestation (Figure 2) at *pI* 4–7, whereas the remaining spots were detected with *pI* > 7.0 (figure not shown). Mixed models ANOVA using BPH induced proteins in the control and BPH infested IR64 treatments shows that a larger cohort of these proteins was changed only during T3 and T4 stage, indicating higher stress response at the later stage (Figure 3). Since the effect of BPH stress was more evident at T3 (28 DAI), we compared the protein abundance at T3 in isolation using control and BPH infested plants. Comparison of protein abundance (spot volume of infested/control at T3 showed that a total of 36 proteins increased >1.5 fold while 29 proteins showed <0.5 fold decrease with BPH infestation. The protein abundance showed a reduction through time as the plants entered senescence at T4 (34 DAI).

Based on matrix assisted laser desorption ionization time-of-flight (MALDI-TOF) and quadrupole time-of-flight (Q-TOF) mass spectrometry, the identity of 52 proteins was generated; 27 proteins with increased abundance and 25 proteins with decreased abundance (Table 2). Peptide mass of the remaining 13 of total 65 protein spots did not match with any known proteins in the NCBI protein database. These BPH responsive proteins were classified into 11 functional categories [24] of which 39% belonged to energy category, whereas 16% were stress and plant defense related. The identity and function of 20% of BPH responsive protein spots in IR64 are not known. In general, the dominating category of BPH affected the functional group involved photosynthesis and metabolism related proteins. BPH induced proteins related to photosynthetic processes were identified as Rubisco activase (Ract), various rubisco large subunits, ferredoxin [(flavodoxin-NADP(H)] reductase (FNR) and oxygen evolving enhancer protein 3 (OEE3) in IR64. This indicates that photosynthesis was one of the common responses to BPH infestation. Likewise, oxidative stress response proteins such as ascorbate peroxidase (APX), GSH dependent dehydro-ascorbate reductase, and CuZn superoxide dismutase (SOD) were identified as BPH stress response proteins in IR64. Abundance of multiple spots of ribulose bisphosphate carboxylase large (rubisco, rbcl) subunits (4 spots), ascorbate peroxidase (APX) (5 spots), unnamed protein (2 spots), oxygen evolving enhancer protein 3 (2 spots), and enolase (2 spots) may represent post translational modifications during BPH stress or presence of multiple gene copies of these proteins in rice.

**Figure 4:**
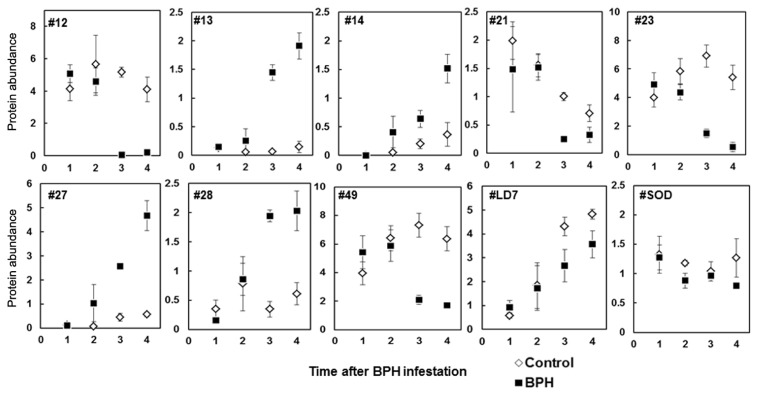
Relative protein abundance of brown planthopper (*N. lugens*) altered stress- and defense-related proteins in BPH infested and control IR64 at different days after infestation (DAI) (T1 = 2 DAI; T2 = 13 DAI; T3 = 28 DAI; T4 = 34 DAI). The protein abundance was quantified with Melanie3 software. Mixed models ANOVA was used for repeated measures analysis of proteins. Mean ± SE (*n* = 3).

Abundance of several oxidative stress-response proteins, drought (#LD7) and two salt stress (#23 and #27) response proteins was altered with BPH stress as observed at T3 (Figure 4). Repeated measures analysis with individual spot abundance in control and BPH infested plants indicated that the spots #13, #14 and #28 were consistently increased (*p* < 0.05) with BPH stress over time whereas spots #12, #21, #23, #49 and #LD7 showed significant decrease as compared to the control (*p* < 0.05) over time (Figure 4). Although the protein “#LD7” (S-like RNase) was less changed with BPH infestation as compared to the control plants, protein levels increased through time during infestation (*p* < 0.05). The abundance of protein spots #23 and #27, which showed similarity to salt stress root protein “RS1” (Gi34904362) [25], also changed differentially with BPH infestation at different times, particularly at T3 and T4 (*p* < 0.05). At T3, the abundance of protein #23 decreased > 2 times (*p* < 0.05) than in control plants, while the protein spot #27 which remained suppressed in control plants, was however more abundant with infestation through all four time points (Figure 4).

### 2.3. Rice Proteins Induced in BPH Infested Plants

**Figure 5:**
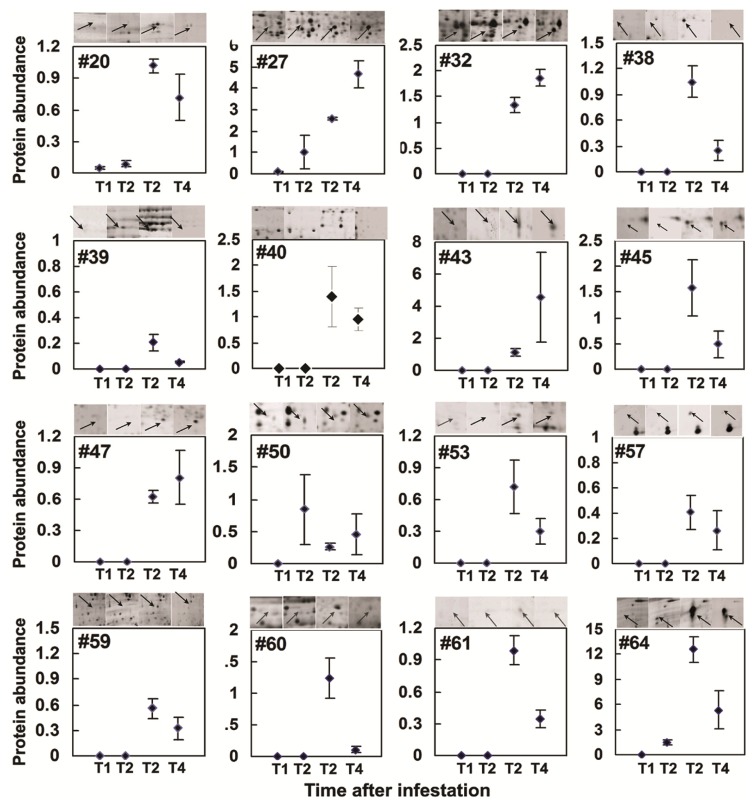
Relative abundance of brown planthopper (*N. lugens*) responsive proteins in IR64 at different days after BPH infestation (DAI) (T1 = 2 DAI; T2 = 13 DAI; T3 = 28 DAI; T4 = 34 DAI)). Mean ± SE (*n* = 3).

The abundance of 16 protein spots (spot #20, #32, #38, #39, #−39a, #40, #43, #45, #47, #50, #53, #57, #59–61 and #64) was observed (Figure 5) at different time points only in BPH infested plants. Interestingly, a change in the protein levels of the spot #20 (proteophosphoglycan, PPG), spot #50 and spot #64 (EFTu1) was also observed at T1 and or T2 indicating that these proteins accumulate in IR64 during early BPH-induced stress (Figure 5). Induction of proteophosphoglycan (#20), putative 1,4-benzoquinone reductase (#47), Putative defective chloroplasts and leaves (DCL) protein (#53), Putative FH protein NFH2 (#59), hypothetical protein P0677B10.12 (#60), putative oxygen evolving enhancer protein 3-1, chloroplast precursor (#61) and chloroplast translation elongation factor Tu1 (#64) have not been reported earlier in BPH-rice interactions and may have role in rice resistance to BPH infestation. The highest levels of these proteins was observed with spot #64 (spot density = 12.58 ± 1.52) at T3 as compared to the abundance of other proteins whereas the spot #39 (0.20 ± 0.06) was least induced with BPH infestation. The abundance of all these BPH induced proteins, except spots #32 (enolase), #43 (unknown), and #47 (putative 1,4-benzoquinone reductase) showed declining trend at T4 as the plants started to senesce.

A few proteins identified in this study were also non-rice proteins (#38, #39, #39a and #68). Spot #38 was identified as “ATP-dependent DNA helicase UvrD (*Shewanella denitrificans* OS217)”. Also #39a with molecular weight of 97.5 kDa showed similarities to leech derived protease inhibitor protein (LDPI) and #39 showed a similarity with “Vitellogenin” from BPH. Spot #68 matched to “Succinyl-CoA ligase [ADP-forming] subunit beta OS = *Mesorhizobium* sp. (strain BNC1)”. These proteins could be either BPH associated proteins injected into rice sheath during feeding or environmental contaminants that colonized BPH wounded rice plants.

### 2.4. Comparative Proteomics of IR64 and Mutants

To understand the defense response of rice against BPH infestation, the protein levels in control and BPH infested IR64 were compared with gain (D518) and loss of resistance (D1131) mutants of IR64 at T3. These mutants were previously identified during a screening of chemically generated IR64 mutants against BPH using a modified seedbox screening technique [23]. Field performance of these mutants did not show compromise in agronomical traits due to mutations.

By comparing the protein abundance (protein volume in BPH infested/control) between IR64 and the mutants, 22 proteins were identified that showed differential abundance (Table 3). Ten proteins were altered in a unique manner in the susceptible mutant (D1131) when compared to IR64 and resistant mutant (D518). Among these proteins, eight proteins (spot #7, #43, #45, #47, #53, #57, #59, #B) were significantly increased (*p* < 0.05) whereas two proteins (#21 and #32) were highly decreased in D1131 (*p* < 0.05) than in D518 and IR64. The protein #27 generally increased with BPH stress, however showed little change in D1131 whereas comparatively, the abundance of this protein was in greater quantities in IR64 and D518 following BPH infestation (*p* = 0.018). In contrast, twelve proteins were linked to a D518 related response to BPH (Table 3). Three proteins (#35; #38 and #40) were significantly (*p* < 0.05) reduced in D518 during BPH stress; another five proteins (#9, #21; #29, #30 and #31) were least affected in D518 whereas the same proteins were decreased in IR64 and D1131 (*p* < 0.05). Similarly, two proteins (#27 and #LD7) showed higher levels (*p* < 0.05) in D518 as compared to IR64 and D1131 The abundance of protein #64 was higher than D1131 but this difference was not significant than IR64. Two proteins (#8 and #41) though increased in abundance, but to a lesser extent (*p* < 0.05) in D518 compared to IR64 and D1131. The abundance of spot “LD7” exceptionally increased in D518 but reduced in IR64 and D1131 with BPH stress. When compared over time after BPH infestation, the protein spot #LD7 remained unchanged at T1 and T2, increased to greater quantities at T3 and decreased thereafter at T4 in D518.

**Figure 6:**
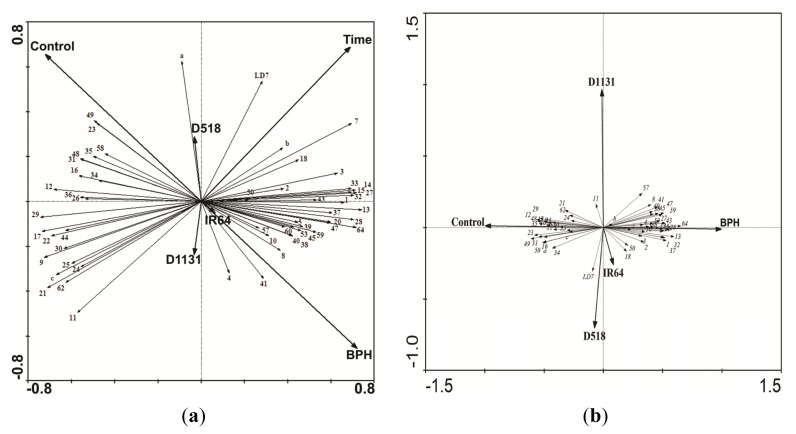
Redundancy analysis (RDA) biplot of protein abundance over the duration of the experiment. All factors are illustrated as thick vectors and include Control, Treatment (BPH infested), Loss of resistance (D1131), Gain of Resistance (D518), Wild type (IR64), and Time. Proteins are illustrated as thin vectors and consist of the proteins levels which are listed as a number as described in Table 2. Eigen values (lambda) are 0.324, 0.050, 0.010, and 0.004 using data at T3 (**a**) and all 4-time points (**b**) Monte Carlo test (1000 permutations) for all canonical axes: F-ratio = 8.490, *P* = 0.001.

From the biplot analysis, it is clear that the variation in the levels of specific proteins was associated with specific factors. For example, the variation in the abundance of proteins #12, #29, #23, #35, #48 and #49 were associated with the control. Furthermore, all proteins whose eigenvectors are travelling in the same direction as the thick eigenvectors, are associated with that factor. Likewise, #LD7 was associated with D518 and to a lesser extent IR64. Several proteins including #64, #28, #13, #32 were associated with the “BPH infested” treatment. Variability in the protein 11 (unknown protein) was the only one clearly associated with D1130 (Figure 6a). Similarly, broader random experimental factors can be included to evaluate responses to covariates (Figure 6b). Variation in the abundance of proteins located on the right side of the biplot (Figure 6b) indicates that change in protein levels was associated with the progression of “time” and the presence of “BPH infestation”, whereas proteins on the left side of the biplot were associated with the lack of treatment or control as well as the earlier time points. Interestingly, levels of protein #a (unknown protein) was strongly associated with D518 and conversely, variation in the induction of protein #4 (Putative ribosomal protein s12) was associated to a lesser degree with D1131 and to a greater degree BPH infestation. IR64 did not explain a significant proportion of variation in the protein abundance data.

## 3. Discussion

Rice resistance to brown planthopper (BPH) is intricate involving genetically controlled defense mechanisms. Despite the existing knowledge of a large collection of rice genes, the molecular response involved in rice stress physiology particularly during interactions with BPH remained elusive. Mutants are valuable source of genetic diversity for gene discovery that could provide valuable information to explain plant defense mechanisms [18–20,26]. We used mutants of the indica rice IR64 that differ in their response to BPH infestation to facilitate the understanding of rice resistance mechanisms to this economically important pest of rice. The time dependent differential change in the levels of BPH response proteins in rice helped to discriminate wild type with the mutants and revealed candidate proteins involved in plant resistance against BPH infestation.

Initially, the response of wild type IR64 was determined during BPH infestation, and proteins related to various functional categories were identified in BPH infested IR64; nevertheless photosynthesis, metabolism, and oxidative stress related proteins were predominantly altered (Table 2). It has been reported that BPH infestation reduces photosynthetic activity in rice due to excessive loss of plant assimilates, decreased leaf area and wilting [11,27]. Phloem feeding insects are generally known to alter the expression of genes required for photosynthesis [14,28]. However, the role of housekeeping proteins such as those related to photosynthesis cannot be ruled out in defense against insects as housekeeping genes could shift their role towards defense metabolism to manage the increased energy demands during stress [29,30]. For instance, photosynthesis-related genes altered during plant-insect interaction contributed towards defense needs while protecting the basic photosynthetic capacity [29,30]. We also found a number of Rubisco large subunit fragments (RLSU) with BPH infestation. Similar observations have been reported with abiotic and biotic stresses in rice [31,32]. Presence of several Rubisco large fragments (rbcl) with various experimental molecular weights and *pI*s could also be due to oxidative stress induced fragmentation of the major Rubisco protein which is an abundant source of macronutrients such as nitrogen in senescing leaves [31,33,34]. This supply of nitrogen during stress might serve as fuel for metabolic processes increased during BPH feeding stress.

We also observed changes in the levels of several antioxidant proteins that are known to scavenge excessive reactive oxygen species generated under stress [9,31,35,36]. Some of these oxidative enzymes can be antinutritive to insects [37,38]. Increased levels of oxidative enzyme activity might have adverse effect on the BPH performance thus helping to reduce damage. Similarly, generation of ROS can also act as stress signals to induce defense related genes during insect infestation [39]. Few ascorbate peroxidase (APX) isoforms were found to be induced as early as 13 DAI (Figure 4), indicating their primary importance during BPH infestation and implication in defense signaling. Moreover, we observed differential levels of APX related proteins in BPH infested IR64 as three of the APXs were increased whereas two were decreased during the infestation which is in agreement with previous studies on differentially induced ascorbate peroxidase isozymes during oxidative stress [40].

Induction of proteins during stress is important in dealing with the stress-induced metabolic homeostasis through readjusting metabolic pathways and reallocation of plants’ resources for defense [41–43]. During such response, proteins may be reduced or increased in activity as evidenced in this study. We observed 64 proteins induced with BPH infestation and 52 of these were identified (Table 2), some of these might have role in higher energy demands during stress. This seems plausible as many of these proteins (Table 2, Figures 4 and 5), except for few non-rice proteins (#20, #38, #39, #39a, #40, #41, #68), are plant stress response proteins. These induced proteins could be by-products of stress metabolism or post translation modification but may also represent molecules needed in signal transduction or acclimation response of plants during stress [42]. Fifteen proteins (Figure 5) were observed only in BPH infested plants whereas these proteins were absent in controls. BPH induced proteins, some of which are still unknown, are potentially involved in rice defense during BPH stress. Induction of several other proteins (#23, #27 and #LD7) during BPH stress showed rice response similar to that observed in abiotic stress such as drought and salinity [25]. Excessive loss of phloem sap and impaired water movement during BPH infestation leads to wilting like condition “hopperburn” which is the susceptible response of rice to BPH [9,11]. Phloem feeding insects generally reduce foliar water potential in plants as a result of extensive feeding and results in the induction of transcripts associated with water stress [28,44,45]. Any counter activity such as altered levels of abiotic stress related proteins that could to delay wilting may help to overcome BPH stress. Up-regulation of drought induced S-like RNase and salt stress induced proteins in BPH infested rice points the need for exploring these proteins in rice defense response to BPH stress.

Comparative analysis was performed to differentiate the proteome response of mutants from the IR64. Defensive response of mutants was demonstrated by differential pattern of proteins induced with BPH infestation. For example, abundance of stress induced glyoxalase I, known with plant defense activity [46], was reduced in D1131 and IR64 but not to the same extent in D518 (Table 3). A similar response was evident with GSH-dependent dehydro ascorbate reductase in D518. The protein EFTu1, similar to 45- kDa heat shock proteins with chaperone like activity [47,48], was induced earlier (T2) and more intensely in D518 and IR64 (S Figure 1) and its abundance was greater in D518 followed by IR64 and then D1131. EFTu1 has been reported as an important component of thermo-tolerance in maize and other environmental stresses [48]. Another two proteins, S-like RNase and spot #27 were also more abundant in D518 in contrast to moderate levels of these proteins in IR64 and susceptible mutant D1131 (Table 3). Higher levels of these proteins in D518 could be important in providing defense to D518 against increasing BPH stress. Similarly, abundance of certain proteins was highly reduced in D518 during BPH infestation whereas the decrease in protein levels was slow in IR64 and D1131 suggesting for higher metabolic shift or adjustment of metabolic pathways in the resistant mutant. On the contrary, some proteins were in greater quantities in D1131 than IR64 and D518 and may represent a susceptible response during BPH infestation (Figure S1). Several antioxidant enzymes and their isoforms were affected with BPH stress. Differential modulation of antioxidant proteins in a resistant and susceptible rice line infested with BPH was previously reported [9]. However, we could not differentiate IR64 resistance solely from its mutants based on antioxidant proteins such as APX as levels of these proteins were not different.

Differential induction of drought induced S-like RNase and salt stress induced proteins (spot #23 and #27) suggests for the relationship between rice resistance to BPH and abiotic stress that urges for exploring abiotic stress tolerant varieties against BPH and vice versa. S-like RNase genes constitute an important family of RNA-degrading enzymes that have been associated with phosphate starvation, ethylene responses, senescence and programmed cell death and defense against multiple stresses [25,49–51]. Sticky digestive liquid from a carnivorous plant, *Drosera adelae*, contained an abundant amount of S-like RNase which assists plants to obtain phosphates from trapped insects which help to defend them against microbes [52]. Induced S-like RNase has shown to prevent the growth of fungal hypha in tobacco [53]. It is likely that increased abundance of S-like RNase may play a role to protect the resistant cultivar D518 from BPH perhaps by inhibiting stylet or ovipositor movement in phloem sheath and reduced settling, feeding and egg laying has previously been observed [23]. Further studies in this area will elucidate mechanisms that S-like RNase and other proteins might play in rice resistance to BPH. One option is to investigate the interaction of BPH induced rice proteins with *in silico* structure analysis and molecular docking (to reveal complexity of rice response to BPH stress particularly for possible links to phosphate (Pi) starvation, plant-microbe interaction and drought. Further experiments with *in silico* and transgenic approach will help to elucidate the precise role of BPH induced proteins in rice defense to BPH.

## 4. Experimental Section

### 4.1. Insect Culture and Plant Material

Brown planthopper (BPH), *Nilaparvata lugens* (Stal) populations were continuously maintained on the susceptible variety “Taichung Native 1” (TN1) at the International Rice Research Institute (IRRI), Los Baños, Philippines. The parent BPH population was collected from rice fields around IRRI, Laguna. Gravid females were used to get a synchronized hopper stage for infestation.

The Indica rice cultivar IR64 along with its two mutants, *i.e.*, D518 (gain-of resistance) and D1131 (loss-of-resistance) generated through the chemical and radioactive mutagenesis of IR64 [23] were used for this study. The mutant D518 shows enhanced resistance during BPH infestation whereas D1131 is susceptible. The mutants were used following six generations of selfing and after confirmed field evaluation showing absence of any deleterious effect of mutations. The field trials of these mutants revealed no agronomical differences from IR64 [23] whereas analysis using IR64 specific molecular markers suggested that the mutants are essentially near-isogenic (unpublished data). The experimental plants were maintained under greenhouse conditions at 28 ± 2 °C with a photoperiod of 16 h day/8 h night cycle.

### 4.2. Plant Phenotype to BPH Infestation

Phenotypic response of IR64 to BPH infestation was determined using a modified seedbox screening technique under greenhouse conditions [13]. This technique provides free choice to BPH nymphs to colonize the plants in the seedbox. Briefly, pre-germinated seeds were sown in seedboxes (45 cm × 35 cm ×15 cm) containing heat-sterilized soil in six equally spaced rows (two rows for each entry) and 15 seedlings per row. Each row (mutant or wild type) was randomized within a seedbox and replicated in three independent seedboxes. Ten-day-old seedlings were uniformly infested with 3–4 second-instar BPH nymphs per plant and allowed to settle on plants of their choice. Hopperburn symptoms were observed 34 days after infestation (DAI).

### 4.3. Proteomics Response after BPH Infestation

Since phenotypic response of IR64 differed with two mutants, a no-choice setup was planned to allow equal number of BPH stress to feed on these genotypes. Fifteen seeds of mutants or wild type plants were sown in individual nine inch circular pots using three technical and three biological replicates. The seedlings were maintained in the greenhouse and before infestation with three nymphs per plant 10 days after sowing, pots were randomized between entries and covered with mylar cage and infested. Control plants were not infested but were covered with mylar cage and arranged randomly. For protein extraction, the plants from three experimental and biological replicates were sampled at four time points after infestation. For the first sample (T1), plant tissue was harvested 2 DAI when the infested nymphs were still in 3^rd^–4^th^ instar stage; the second sampling (T2) was done at 13 DAI when the majority of nymphs were at the adult stage; the third sampling (T3) was performed 28 DAI following the emergence of second generation nymphs; the fourth sampling (T4) was done when the susceptible mutant (D1131) started wilting (34 DAI). For protein analysis, a 10 cm sample above ground portion of leaf sheath was harvested and stored immediately in liquid nitrogen. For control, plants were harvested at same time points using non-infested plants.

### 4.4. Protein Analysis

Protein extraction. Total leaf sheath proteins were extracted in a precipitation solution (10% Trichloroacetic acid, 89.93% Acetone, 0.07% Dithiothreitol) using a modified method of Damerval *et al.* [54]. The protein concentration was determined using a Protein-Assay-Kit (Bio-Rad) following the manufacturer’s instructions.

Protein separation and image analysis of 2D Gels. Gel electrophoresis was performed using non-linear (NL) 18-cm IPG strips with pH 4–7 and 3–10 (Amersham Pharmacia Biotech, Uppsala, Sweden). The IPG strips were rehydrated overnight in 350 μL of rehydration buffer and 100 μg of sample protein. The isoelectric focusing (IEF) of proteins was performed on a Multiphor II Electrophoresis unit (Amersham Biosciences) at 20 °C with constant 200 V for the first hour, 500 V for next 2 h and finally 16 h at 2950 V. Proteins from DTT/IAA equilibrated IEF strips were separated on 15% sodium dodecyl sulfate (SDS) polyacrylamide gels using a Protean-II Multi cell (Bio Rad: Hercules, CA, USA) at 4 °C.

The gels were stained with silver nitrate (Sigma Aldrich) for scanning or spot quantification analysis whereas coomassie blue stain (Sigma Aldrich) was used for protein identification with mass spectrometry using standard staining protocols. The gels were scanned with a GS-800 Calibrated Densitometer (Bio-Rad) at a resolution of 600. For spot detection, protein quantification and spot analysis, Melanie-3 image analysis software (GeneBio, Geneva, Switzerland) was used. Spot detection parameters were as follows: number of smooths, 5; Laplacian threshold, 5; partial threshold, 1; saturation, 90; peakness increase, 100; minimum perimeter, 10. The Melanie software automatically normalized the spot intensity (the relative volume) *i.e.*, the volume divided by the total volume over the whole image (Melanie 3 user manual). The percent spot volume detected by software was used to match spots for intensity differences and predict BPH induced proteins.. The protein spots were categorized as BPH altered (increased or decreased in abundance) if protein abundance in a rice line increased or decreased with BPH infestation compared to mean control value. Abundance ratio (protein volume in infested plants/control plants) was compared with control at a time point to determine fold change in proteins. An arbitrary cutoff was used to express highly altered proteins [>1.5 (increased), <0.5 (decreased) or >0.5 and <1.5 (least altered)].

### 4.5. Protein Identification

The proteins spots from Coomassie Brilliant Blue (G-250) stained gels were manually excised using a sterilized scalpel and submitted to the Australian Proteome Analysis Facility (APAF) Macquarie University, Sydney, Australia [55] for characterization. Protein samples were analyzed with matrix assisted laser desorption ionization time of flight (MALDI-TOF) mass spectrometry using a Micromass Tofspec time-of-flight mass spectrometer (Micromass, Manchester, UK) at APAF following standard procedures. If proteins could not be identified with MALDI-TOF, a further analysis was performed on Q-TOF LCMS. For protein identification, peak lists were used and peptide masses were searched against SWISS-PROT and NCBInr databases using the Mascot search engine [56] supported by Matrix Science Ltd., London. In MS/MS Ion Search, following parameters were used for database queries on monoisotopic peptide masses using the Viridiplantae and *Oryza sativa* as taxonomic categories; peptide mass tolerance of 150 ppm; fragment mass tolerance: ±0.6 Da; variable modifications: Oxidation (M), Propionamide (C); and the maximum number of missed tryptic cleavages, 1. Peptide masses that yielded a significant ion score (*p* < 0.05) were considered positively identified.

### 4.6. Statistical Analysis

Data analysis was performed with Statistical Analysis Software (SAS) (Version 9.1) and JMP-IN (Version 5.1) (SAS Institute, Cary, NC, USA) using protein abundance values in control and BPH infested plants of three genotypes (wild type IR64 and two mutants) and compared at each respective time point. Protein abundance ratio in relation to each control group (IR64 or mutants) was calculated by dividing the spot abundance in the BPH infested plants by the mean spot abundance of the control plants and expressed as fold change with statistical significance at p-value lower than 0.05. A 2-way ANOVA was used to compare the protein abundance between IR64 and the mutants and the means were separated with the Tukey’s HSD multiple means comparison test (*p* < 0.05). Ordination statistics were performed on protein abundance and genotypes to measure interactions between the BPH and rice proteins (Canoco V.4.5) [57]. Initially, detrended correspondence analysis (DCA) was performed to measure eigenvector length of expressed proteins variables (control, infested) [26]. Redundancy analyses (RDA) were performed and the significance of the first two axes, as well all four axes, were tested using a Monte Carlo test with 1000 permutations in reduced space. The reason RDA was chosen in this particular instance rather than another multivariate method, is that the variable data showed *linear* responses as opposed to *unimodal* responses. Multivariate biplots allow one to explore trends through numerical data analysis above and beyond simple hypothesis testing. Where relationships and covariation between variables is not evident with simple univariate statistics, multivariate methods clearly show the abundance of specific proteins as variables in relation to experimental factors. In this case it is clear that specific proteins covary with specific treatments, and the treatments themselves also show covariation.

## 5. Conclusions

**Figure 7:**
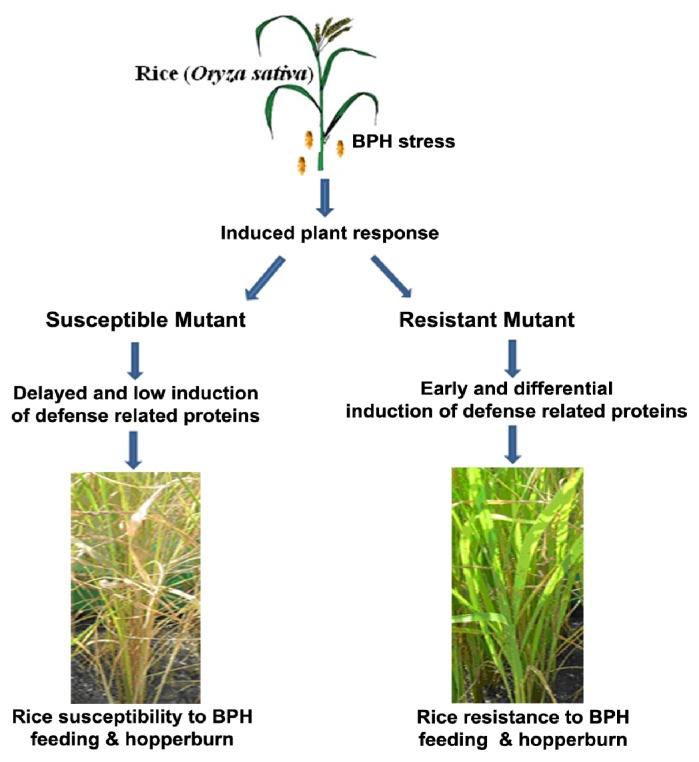
A summarized figure of brown planthopper (*N. lugens*) induced IR64 proteins. Abundance of various proteins associated with rice resistance is altered following BPH infestation. The resistant lines such as D518 may induce specific genes earlier and more intensely than susceptible lines that interact with other proteins thus leading to their enhanced level of resistance against BPH.

BPH infestation on rice cv. IR64 altered the induction of several proteins involved in various functional categories. A differential induction in proteins was evident both in resistant and susceptible mutant of IR64. Overall, D518 essentially resists against BPH attack via increased activity of proteins related to metabolism (Glyoxalase I, Probable ATP synthase 24 kDa subunit, Enolase), stress response (S-like RNAse, GSH-dependent dehydro ascorbate reductase, Salt stress root protein “RS1”) and protein synthesis (Chloroplast translation elongation factor Tu1) (Table 3). Altered abundance of proteins, in particular lower levels of stress related proteins might have role in susceptibility of D1131 (Table 3). Moreover, the resistant plant also appears to compensate through a timely induction of some of these proteins thus providing a leading edge over the susceptible plants. Differential response of the mutants to BPH feeding thus leads to altered hopperburn symptoms on the rice plants (Figure 7). The complex plant response to BPH also insists on refocusing the research for rice defense towards other metabolic pathways like photosynthesis and their possible interaction to understand rice resistance mechanisms to BPH infestation and to develop resistance breeding program. Further experiments to explore a defined biological interaction between differentially induced proteins with other housekeeping proteins may explain how resistant mutant would overcome BPH stress than susceptible mutant D1131 or moderately susceptible IR64.

## Figures and Tables

**Table 1:** Comparative reaction of IR64 and mutants to brown planthopper (BPH) infestation at different times (T1 = 2 days; T2 = 14 days; T3 = 28 days; T4 = 34 days). The infested plants were observed for BPH feeding damage and rated using a 1–9 scale (1 = Resistant, no damage symptoms; 3 = Slight damage, pale outer leaves; 5 = wilting on 50% leaves, slight stunting; recovery possible if insects removed; 7 = Severe hopperburn, only one or two leaves green, no recovery possible; 9 = Highly susceptible, complete wilting). (*n* = 15, Mean ± SE).

Rice line	BPH damage (1–9 scale)			

	T1	T2	T3	T4
IR64	1.0 ± 0.0	1.6 ± 0.55	3.6 ± 0.55	5.2 ± 0.85
D518	1.0 ± 0.0	1.4 ± 0.48	3.0 ± 0.76	3.6 ± 0.56
D1131	1.0 ± 0.0	1.8 ± 0.59	4.8 ± 0.65	6.8 ± 0.66

**Table 2:** List of 52 leaf sheath proteins induced during BPH stress on rice variety IR64.

Spot	PM (%C)	Identity/source	Accession	Exp. (Theo.) *M*_r_	Exp. (Theo.) *pI*	Mascot score	Fold change	*P*-value
**Energy/pentose phosphate**								

1	2(4)	Rubisco large subunit	gi11955	17.2(52.8)	4.5(6.13)	64	>10 ↑	0.047
2	1(4)	Rubisco large subunit	gi476752	17.3(45.1)	4.6(8.4)	104	>10 ↑	0.006
3	2(5)	Ribulose bisphosphate carboxylase/oxygenase large chain	gi11466795	17.2(52.8)	5.1(6.2)	98	4.56 ↑	0.008
5	3(9)	Rubisco large subunit from chromosome 10 chloroplast insertion	gi37533338	23.7(52.8)	5.4(6.4)	128	1.53 ↑	0.130
10	5(13)	Rubisco large subunit	gi476752	24.9(45.1)	6.1(8.4)	174	>10 ↑	0.005
RA*	–	Ribulose bis phosphate carboxylase/oxygenase activase	P93431	47(42.07)	5.0(5.0)	–	11.45 ↓	0.0019
Rb	3(9)	Rubisco large subunit	gi2734976	34.1(43.7)	6.3	332	3.35 ↑	0.006

**Energy/photosynthesis**								

61	1(33)	Putative oxygen evolving enhancer protein 3-1 chloroplast precursor	gi50938199	18.7(22.9)	9.8(9.8)	114	Ind ↑	0.0053
34	13 (38)	Ferredoxin-NADP (H) oxidoreductase	Q6ZFJ3_ORYSA	36.0(40.8)	5.9(7.9)	90	4.21 ↓	0.0114
63	3(44)	Putative oxygen evolving enhancer protein 3-1 chloroplast precursor	gi50938199	14.5(22.9)	9.9(9.8)	400	2.57 ↓	0.0053

**Energy/glycolysis**								

32	2(8)	Enolase	gi33113259	37.8(47.9)	5.5(5.4)	77	Ind ↑	0.0009
37	14 (49)	Enolase	gi780372	39.9(47.9)	6.7(5.4)	104	7.95 ↑	0.0522
TP	8(25)	Triose phosphate isomerase, cytosolic	P48494	27.5(27.1)	5.6(5.4)	70	<10 ↓	<0.0001
9	6(33)	Glyceraldehyde-3- phosphate dehydrogenase, cytosolic	G3PC_HORV	37.4(33.2)	6.7(6.2)	258	9.6 ↓	0.0090
44	6(27)	Putative dihydrolipoamide dehydrogenase precursor	gi34894800	57.0(52.6)	6.6(7.2)	111	<10 ↓	0.0011
35	3(17)	Formate dehydrogenase	gi51536124	41.2(41.3)	6.6(6.7)	100	<10 ↓	0.0005
FB*	–	Fructose bisphosphate aldolase	Q40677	37.7(36.4)	5.7(5.8)	–	1.31 ↓	0.1620

**Energy/electron transport**								

58	3(34)	Putative H(+)− transporting ATP synthase	gi50912809	25.5(26.2)	4.3(4.9)	305	3.57 ↓	0.202
30	8(50)	Probable ATP synthase 24 kDa subunit	gi50905037	28.1(27.2)	5.5(6.5)	300	4.89 ↓	0.0007

**Plant defense**								

13	6(32)	L-Ascorbate peroxidase 2, cytosolic *Oryza sativa* subsp. japonica (Rice)	APX2_ORYSJ	26.3(27.1)	5.3(5.2)	65	>10 ↑	0.0005
14	5(36)	Putative ascorbate peroxidase	gi50920595	26.2(27.1)	5.2(5.4)	94	3.16 ↑	0.0604
28	5(39)	Ascorbate peroxidase	gi50940199	28.0(27.1)	5.5(5.2)	239	5.55 ↑	0.0007
49	5(36)	Putative ascorbate peroxidase	gi50920595	22.6(27.1)	6.5(5.4)	71	3.49 ↓	0.0041
12	5(50)	Ascorbate peroxidase	gi50940199	29.1(27.1)	5.2(5.2)	419	<10 ↓	<0.0001
SOD *	–	Superoxide dismutase	P93407	17.7(15.7)	5.8(5.3)	–	1.47 ↓	0.0652

**Stress induced**								

LD7	3(23)	Drought induced S-like RNase protein	gi17105171	28.2(28.4)	5.1(5.2)	187	1.6 ↓	0.1033
27	4(38)	Unnamed protein product (Salt stress induced protein)	gi34904362	29.1(21.8)	4.9(4.9)	153	Ind ↑	0.0003
21 *	–	GSH-dependent dehydro ascorbate reductase	BAA90672	27.0(27.1)	6.1(5.4)	–	4.03 ↓	0.0009
23	3(40)	Unnamed protein product (Salt stress induced protein)	gi34904362	30.3(21.8)	4.9(4.9)	179	4.6 ↓	0.0029

**Protein synthesis**								

64	3(23)	Chloroplast translation elongation factor Tu1	gi50910077	43.7(50.4)	4.3(6.19)	306	Ind ↑	0.0012
4	3(45)	Putative ribosomal protein s12	gi50934241	17.2(14.8)	5.3(5.3)	290	2.64 ↑	0.0390
22	8(34)	Putative ribosome recycling factor, chloroplast precursor	XP_478772.1	26.4(29.7)	6.0(9.3)	66	2.77 ↓	0.0007

**Protein destination and storage**								

CP	6(23)	Putative clp protease	OS02g42290	30.2(31.9)	5.7(6.7)	70	1.77--↑	0.0051
24	3(20)	Putative chaperonin 21 precursor	gi51091339	27.2(25.4)	4.9(5.9)	60	4.58 ↓	0.0148

**Growth and division**								

41	4(20)	(O65316) Actin (*Mesostigma viride*)	ACT_MESVI	68.3(41.5)	5.8(5.3)	181	7.27 ↑	0.0097

**Secondary metabolism**								

47	3(21)	Putative 1,4-benzoquinone reductase	gi34910128	24.7(21.7)	6.3(6.0)	79	Ind ↑	0.0004
26	4(20)	Putative NADPH-dependent mannose 6-phosphate reductase	gi50904895	36.3(35.4)	6.2(5.9)	142	>10 ↓	0.0106
31	4(23)	Glyoxalase I	gi16580747	34.0(32.5)	5.5(5.5)	173	9.12 ↓	0.0004

**Miscellaneous**								

20	9(15)	Putative proteophosphoglycan	gi50918953	74.0(96.8)	4.4 (10.5)	104	Ind ↑	0.0001
53	8(41)	Putative defective chloroplasts and leaves (DCL) protein *Oryza sativa*	Q6UUF7_ORYSA	30.8(21.3)	6.9(9.0)	69	Ind ↑	0.04
59	18 (27)	Putative FH protein NFH2.-*Oryza sativa* (japonica cultivar-group)	Q8S0F0_ORYSA	13.3(10.2)	4.7(8.9)	66	Ind ↑	0.0099
60	4(53)	Hypothetical protein P0677B10.12	Q67VJ8_ORYSA	52.5(12.5)	5.1(9.3)	68	Ind ↑	0.0185
69	12 (44)	Putative glyceraldehyde-3- phosphate dehydrogenase (Phosphorylating) *Oryza sativa*	gi115459078	37.2(36.5)	7.8(7.68)	94	1.75 ↑	0.0341
40	9(40)	hypothetical protein OsJ_015102 [*Oryza sativa*]	gi125591269	70.0(25.3)	6.4(11.0)	66	Ind ↑	0.0185
B	6(24)	hypothetical protein OsJ_012934	gi125589101	32.0(35.2)	6.1(5.3)	76	1.37 ↓	0.0277
39	5(3)	Vitellogenin [*Nilaparvata lugens*]	gi342318865	72.5(22.7)	7.9(8.5)	64	Ind ↑	0.0372
39a	9(12)	Chain E, Leech-Derived Tryptase Inhibitor TRYPSIN COMPLEX	gi3318722	97.5(23.4)	6.7	240	Ind ↑	0.0139
17	2(18)	Putative DREPP2 protein	gi50906969	32.0(24.0)	4.8(4.7)	105	8.47 ↓	0.0003
62	11 (36)	hypothetical protein OsI_021661	Q5Z6P9_ORYSA	47.0(43.0)	4.7(4.7)	109	3.55 ↓	0.0095
38	19 (26)	ATP-dependent DNA helicase UvrD *Shewanella denitrificans* OS217	Q3P3H8_9GAMM	70.0(81.6)	6.6(5.9)	78	Ind ↑	0.0050
42	12 (38)	Os12g0420200 [*Oryza sativa* (japonica cultivar-group)]	gi115488340	68.7(41.5)	6.3(8.5)	135	1.87 ↓	0.0476
68	8(22)	Succinyl-CoA ligase [ADP-forming] subunit beta OS = Mesorhizobium sp. (strain BNC1)	SUCC_MESSB	35.5(42.2)	7.5(5.0)	74	1.59 ↓	0.0327

Notes:

* = Proteins identified by Salekdeh *et al.* 2002 [25]; PM = Peptides matched; %C = Percent coverage; Exp. = Experimental; Theo. = Theoretical; Mr = molecular weight; *pI* = isoelectric point; Ind = Proteins induced only in BPH infested plants.

**Table 3:** Comparative abundance of BPH induced proteins between IR64 and the mutants (D518 and D1131) at 28 DAI (Time 3). Superscript letters indicate significant difference in abundance between IR64 and the mutants. Values with same superscript letters are not different (*p* > 0.05), (*n* = 3, Mean ± SE).

Protein	Spot	D518	D1131	IR64	*Prob.* > *F*
Unknown	7	1.06 ± 0.07 ^b^,*	2.18 ± 0.30 ^a^,^▲^	1.17 ± 0.14 ^b^,*	0.014
GSH-dependent dehydro ascorbate reductase	21	0.81 ± 0.11 ^a^,^▼^	0.47 ± 0.12 ^b^,^▼▼^	0.73 ± 0.01 ^a^,^▼^	0.059
Enolase	32	0.77 ± 0.19 ^a^,^▼^	0.31 ± 0.12 ^b^,^▼▼^	1.24 ± 0.15 ^a^,*	0.011
Unknown	43	1.04 ± 0.20 ^b^,*	1.81 ± 0.11 ^a^,^▲^	1.10 ± 0.21 ^b^,*	0.042
Unknown	45	1.68 ± 0.34 ^a^,^b^,^▲^	3.46 ± 0.67 ^a^,^▲▲^	1.58 ± 0.54 ^b^,^▲^	0.085
Putative 1,4-benzoquinone reductase	47	0.89 ± 0.19 ^b^,*	1.85 ± 0.20 ^a^,^▲^	0.62 ± 0.05 ^b^,^▼^	0.004
Putative defective chloroplasts and leaves (DCL) protein *Oryza sativa*	53	0.67 ± 0.19 ^b^,^▼^	1.76 ± 0.17 ^a^,^▲^	0.72 ± 0.25 ^b^,^▼^	0.018
Unknown	57	0.51 ± 0.06 ^b^,^▼^	3.10 ± 1.42 ^a^,^▲^	0.58 ± 0.13 ^b▼^	0.008
Putative FH protein NFH2 *Oryza sativa* (japonica cultivar-group)	59	0.61 ± 0.06 ^a^,^▼^	1.14 ± 0.10 ^b^	0.55 ± 0.12 ^b^,^▼^	0.011
Hypothetical protein OsJ_012934	B	1.18 ± 0.09 ^b^,*	1.67 ± 0.10 ^a^,^▲^	0.74 ± 0.07 ^c^,^▼^	0.002
S-like Rnase	LD7	2.07 ± 0.37 ^a^,^▲^	0.37 ± 0.07 ^b^,^▼^	0.62 ± 0.15 ^b^,^▼^	0.005
Unknown	8	2.58 ± 0.42 ^b^,^▲^	9.15 ± 1.08 ^a^,^▲▲▲^	9.71 ± 2.44 ^a^,^▲▲▲^	0.030
Glyceraldehyde-3-phosphate dehydrogenase, cytosolic	9	0.27–0.05 ^a^,^▼▼^	0.11 ± 0.01^b▼▼▼^	0.10 ± 0.02 ^b^,^▼▼▼^	0.024
Salt stress root protein “RS1”	27	4.09 ± 0.24 ^a^,^▲▲^	1.95 ± 1.36 ^b^,^▲^	2.75 ± 0.17 ^a^,^b^,^▲^	0.018
Unknown	29	0.37 ± 0.05 ^a^,^▼^	0.19 ± 0.03 ^b^,^▼▼▼^	0.08 ± 0.02 ^b^,^▼▼▼^	0.008
Probable ATP synthase 24kDa subunit	30	0.43 ± 0.01 ^a^,^▼^	0.18 ± 0.02 ^b^,^▼▼▼^	0.20 ± 0.02 ^b^,^▼▼▼^	0.061
Glyoxalase I	31	0.94 ± 0.09 ^a^,*	0.47 ± 0.07 ^a^,^b^,^▼▼^	0.33 ± 0.01 ^b^,^▼▼^	0.087
Formate dehydrogenase	35	0.46 ± 0.09 ^b^,^▼^	1.28 ± 0.17 ^a^,*	1.16 ± 0.21 ^a^,*	0.015
ATP-dependent DNA helicase UvrD *Shewanella denitrificans* OS217	38	0.49 ± 0.15^b^,^▼^	1.39 ± 0.21^a^,^▲^	1.04 ± 0.32 ^a^,*	0.011
Hypothetical protein OsJ_015102	40	0.33 ± 0.07 ^b^,^▼^	1.20 ± 0.12 ^a^,*	0.99 ± 0.29 ^a^,*	0.041
(O65316) Actin (*Mesostigma viride*)	41	1.81 ± 0.36 ^b^,^▲^	5.94 ± 1.08 ^a^,^▲▲^	7.27 ± 1.33 ^a^,^▲▲^	0.021
EFTu1	64	14.05 ± 1.49 ^a^,^▲▲▲^	7.78 ± 0.83 ^b^,^▲▲^	12.58 ± 1.52 ^a^,^▲▲▲^	0.065

* No significant change in protein abundance compared to 1 (protein volume in BPH infested/control); (^▲^) Increase in protein abundance; (^▲▲▲^) Highly increased in abundance; (^▼^) Decrease in protein abundance; (^▼▼▼^) Highly decreased in abundance.

## Supplementary Files

Supplementary File 1 Supplementary Information (PDF, 243 KB)
