# Supplementary material for: Proteome Analysis of Rice (Oryza sativa L.) Mutants Reveals Differentially Induced Proteins during Brown Planthopper (Nilaparvata lugens) Infestation
Source: Int J Mol Sci. 2013 Feb 15;14(2):3921–45. doi: 10.3390/ijms14023921 (PMC3588078; doi:10.3390/ijms14023921)
Supplement: Supplementary File 1 — Supplementary Information (PDF, 243 KB) [file ijms-14-03921-s001.pdf]

Supplementary Information

Figure S1. Differentially induced leaf sheath proteins in IR64 compared with the two mutants (D518 and D1131 following brown planthopper (*N. lugens*) infestation at T3 with potential role in the response of D518 (**A**) and D1131 (**B**) during BPH stress.

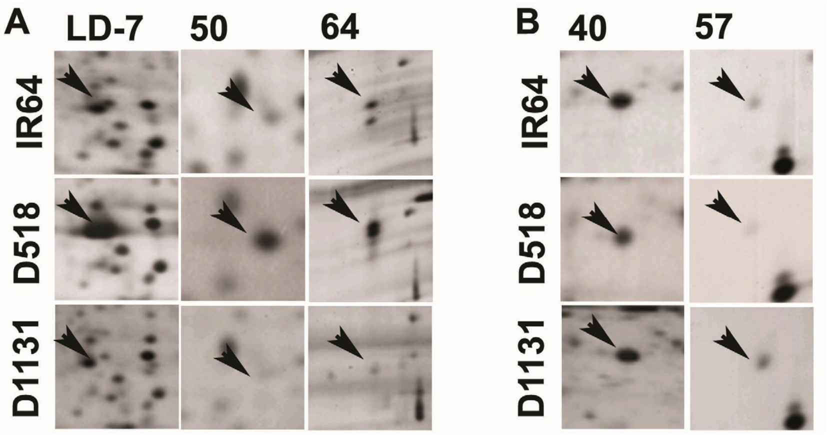

© 2013 by the authors; licensee MDPI, Basel, Switzerland. This article is an open access article distributed under the terms and conditions of the Creative Commons Attribution license (http://creativecommons.org/licenses/by/3.0/).
